# Representation of medical specialties in dean’s cabinets at the top 40 NIH-funded United States medical schools

**DOI:** 10.1186/s12909-025-08548-y

**Published:** 2026-01-05

**Authors:** Anagha Balaji Thiagarajan, Luke Horton, Ajay Nair Sharma, Nathan W. Rojek

**Affiliations:** 1 Department of Dermatology, University of California Irvine School of Medicine, 118 Medical Surge I, Irvine, CA 92697 USA; 2 Department of Dermatology, Medical University of South Carolina, Charleston, SC USA; 3 Department of Dermatology, Massachusetts General Hospital, Harvard Medical School, Boston, MA USA

**Keywords:** Representation, Medical specialties, Dean’s cabinet

## Abstract

**Background:**

Academic leadership within medical schools shapes institutional culture, policy, and educational priorities. While demographic diversity has been studied, specialty representation in leadership is less understood. This study evaluates the distribution of medical specialties in dean’s cabinets at the top 40 National Institutes of Health (NIH)-funded United States medical schools compared with national and hospital-based physician workforce data.

**Methods:**

Publicly available institutional websites were reviewed to identify dean’s cabinet members across eight common leadership roles. Degree type and specialty (for MD/DO holders) were obtained from institutional profiles. Specialty representation was compared with 2023 national and hospital-based physician workforce data using paired t-tests.

**Results:**

Of 320 leaders identified, 287 (89.7%) held an MD or DO. Internal Medicine (IM) accounted for 46.0%, followed by Pediatrics (13.2%) and Obstetrics/Gynecology (5.6%). Compared with the national workforce, IM (*p* < 0.0001), Pediatrics (*p* = 0.006), and Neurosurgery (*p* = 0.003) were overrepresented, while Family Medicine (FM; *p* < 0.0001), Emergency Medicine (*p* = 0.04), Anesthesiology (*p* = 0.008), Orthopedic Surgery (*p* = 0.016), and Ophthalmology (*p* = 0.037) were underrepresented. Similar trends were observed against hospital-based workforce data, with additional overrepresentation of Dermatology (*p* = 0.0008), ENT (*p* = 0.0005), and Plastic Surgery (*p* < 0.00001). Among IM leaders, 41.4% were generalists and 58.6% subspecialists, most commonly in Cardiology, Infectious Disease, and Pulmonary/Critical Care.

**Conclusions:**

Dean’s cabinets at top NIH-funded medical schools are dominated by IM and Pediatrics, with primary care, acute care, and procedural specialties underrepresented. These disparities may influence curriculum design, mentorship, and educational priorities related to community-based care, interprofessional education, interdisciplinary collaboration, and specialty-specific career pathways. Leadership development initiatives targeting underrepresented specialties could diversify expertise in academic medical governance.

## Background

Academic leadership within medical schools is pivotal in shaping institutional culture, policy, and priorities. The composition of leadership teams, such as the dean’s cabinet, not only reflects institutional values but can also influence strategic planning, access to mentorship, and educational opportunities for both faculty and students. Understanding which specialties dominate leadership roles in key functional areas can provide insight into how medical institutional priorities are shaped, and which specialties may be better positioned to influence curricular and policy development. Despite growing awareness of disparities (e.g. gender, race, degree diversity) in medical education, little is known about how medical specialties are represented across high-level academic leadership roles [1–3]. 

Given the increasing unmet need for primary care physicians, it is essential to examine the representation of medical specialties in decision-making positions, particularly at the nation’s most research-intensive medical schools, which often shape the broader climate of medical education and influence national priorities [4, 5]. This gap is particularly relevant as medical schools strive to increase transparency, promote inclusive leadership, and ensure that institutional priorities align with the diverse specialties that constitute modern medicine. By analyzing the medical specialties of dean’s cabinet members at the top 40 National Institutes of Health (NIH)- funded United States (U.S.) medical schools and comparing them to the national physician workforce data, this study characterizes patterns of medical specialty representation across core leadership roles in top medical institutions to inform future leadership development and equity initiatives [6].

## Methods

A review of publicly available institutional websites was conducted to identify dean’s cabinet members of the top 40 NIH-funded medical schools. The top 40 U.S. medical schools were selected based on total NIH funding as reported by the Blue Ridge Institute for Medical Research (BRIMR), in order to focus the analysis on highly research-intensive academic medical centers with comparable institutional missions and leadership structures [6]. 

Dean’s cabinet members were stratified by the top 8 most common leadership titles: Dean, Dean for Student Affairs, Dean for Faculty Affairs, Dean for Clinical Affairs, Dean for Education, Dean for Research, Dean for Admissions, and Dean for Diversity, Equity and Inclusion. These roles were the most consistently represented senior administrative positions across institutions and reflected core functional domains. Alternate titles were considered in data collection as leadership role naming varies by institution (Table 1). When multiple titles existed within a single category, the most senior role was included for analysis.

Members holding an MD, DO, combined degree (e.g. MD/PhD, MD/MBA), PhD, MPH, MHA, or EdD were included in the analysis, and only members holding an MD or DO were included in the analysis of specialty representation. Each member’s specialty was determined using institutional profiles and professional biographies. Individuals explicitly designated as “interim” or “acting” in a leadership role were included if they were listed by the institution as the current occupant of that position. The proportion of dean’s cabinet members in medical specialties identified was compared to the latest available (2023) national physician workforce data and hospital-based workforce data to assess medical specialty representation, utilizing pair t-test analysis in Microsoft Excel [5]. The Association of American Medical Colleges (AAMC) defines the hospital-based physician workforce as those whose primary professional activity occurs within a hospital setting involving direct patient care and working 20 or more hours per week [5]. Hospital-based workforce data was included in this analysis to provide a comparison to a setting most similar to academic medical institutions.

**Table 1:** Alternate role titles for dean’s cabinet members

Standard Role	Common Alternate Titles
Dean	Dean, School of Medicine, Dean of the Medical School
Dean for Student Affairs	Associate Dean for Student/Academic Affairs, Assistant Dean for Student/Academic Affairs, Dean of Students
Dean for Faculty Affairs	Associate Dean for Faculty Development, Assistant Dean for Faculty Affairs
Dean for Clinical Affairs	Vice Dean for Clinical/Medical Affairs, Senior Associate Dean for Clinical/Medical Affairs
Dean for Education	Vice Dean for Education, Senior Associate Dean for Medical Education, Associate Dean for Curriculum
Dean for Research	Vice Dean for Research, Senior Associate Dean for Research/Clinical Research, Associate Dean for Biomedical Research
Dean for Admissions	Associate Dean for Admissions, Assistant Dean of Admissions
Dean for Diversity, Equity and Inclusion	Associate Dean for DEI, Assistant Dean for Diversity, Vice Dean for Inclusion, Dean for Multicultural Affairs

## Results

Of the top 40 NIH-funded medical schools, 320 total dean’s cabinet members were identified.

### Dean’s cabinet members holding an MD or DO

**Fig. 1:**
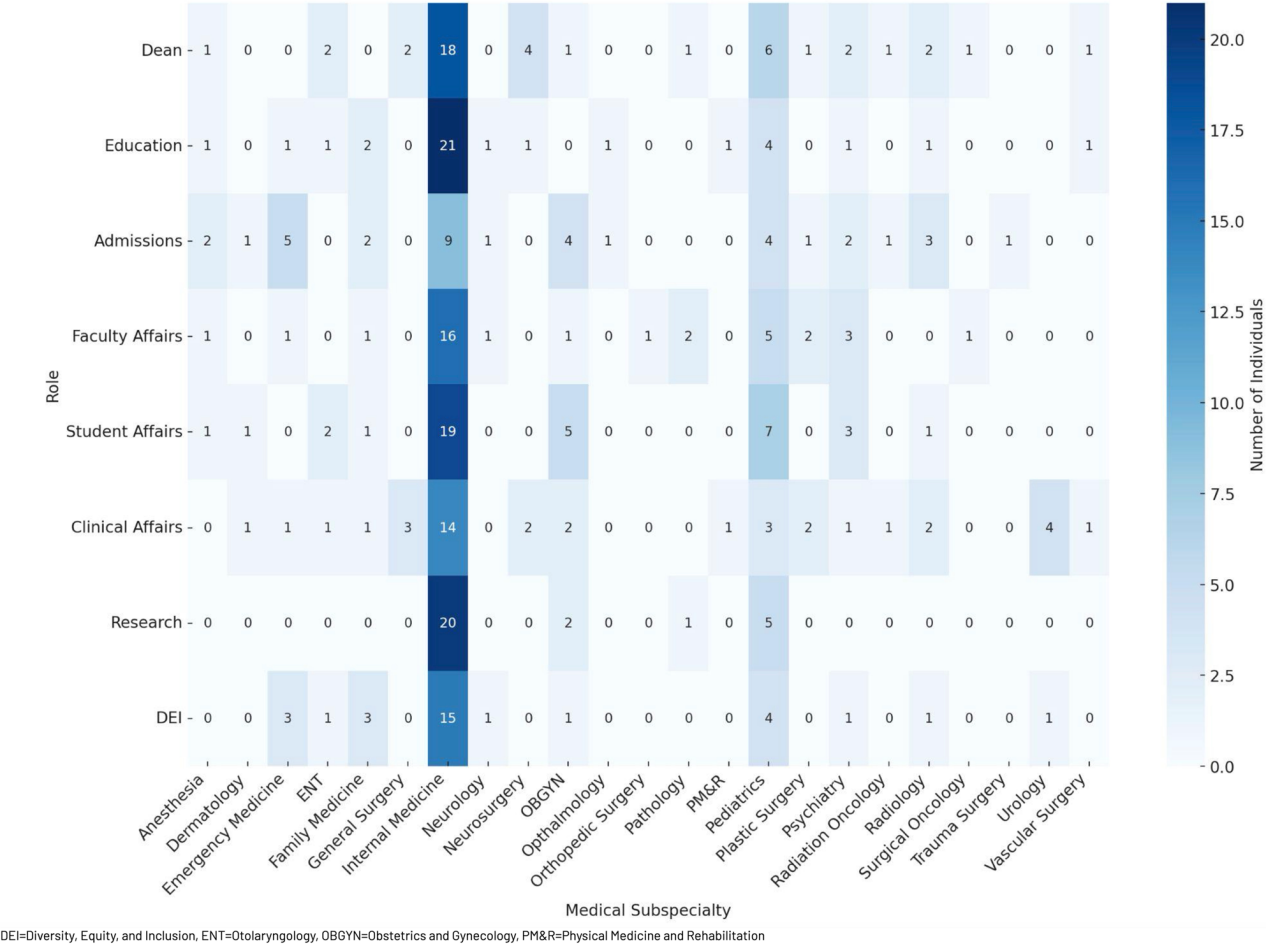
Heat map of represented specialties by dean’s cabinet role

287 (89.7%) members held either an MD or combined degree (e.g. MD/PhD, MD/MBA), with only one member holding a DO degree. Internal Medicine (IM) (132, 46.0%) was the most represented specialty overall, followed by Pediatrics (38, 13.2%), Obstetrics and Gynaecology (OB/GYN) (16, 5.6%), Psychiatry (13, 4.5%), Radiology (13, 4.5%), Emergency Medicine (EM) (11, 3.8%), and Family Medicine (FM) (10, 3.5%). The most common specialty across all dean’s cabinet roles was IM (Table 2). A small number of leadership positions were occupied by individuals designated as “interim” or “acting” at the time of data collection. The distribution of specialties by dean’s cabinet role is further characterized in Fig. 1.

**Table 2:** First and second most represented specialties by dean’s cabinet role

Dean’s Cabinet Role	Most Represented Specialty (#)	2nd Most Represented Specialty (#)
Dean	Internal Medicine (18)	Pediatrics (6)
Dean for Student Affairs	Internal Medicine (19)	Pediatrics (7)
Dean for Faculty Affairs	Internal Medicine (16)	Pediatrics (5)
Dean for Clinical Affairs	Internal Medicine (14)	Urology (4)
Dean for Education	Internal Medicine (21)	Pediatrics (4)
Dean for Research	Internal Medicine (20)	Pediatrics (5)
Dean for Admissions	Internal Medicine (9)	Emergency Medicine (5)
Dean for Diversity, Equity and Inclusion	Internal Medicine (15)	Pediatrics (4)

**Fig. 2:**
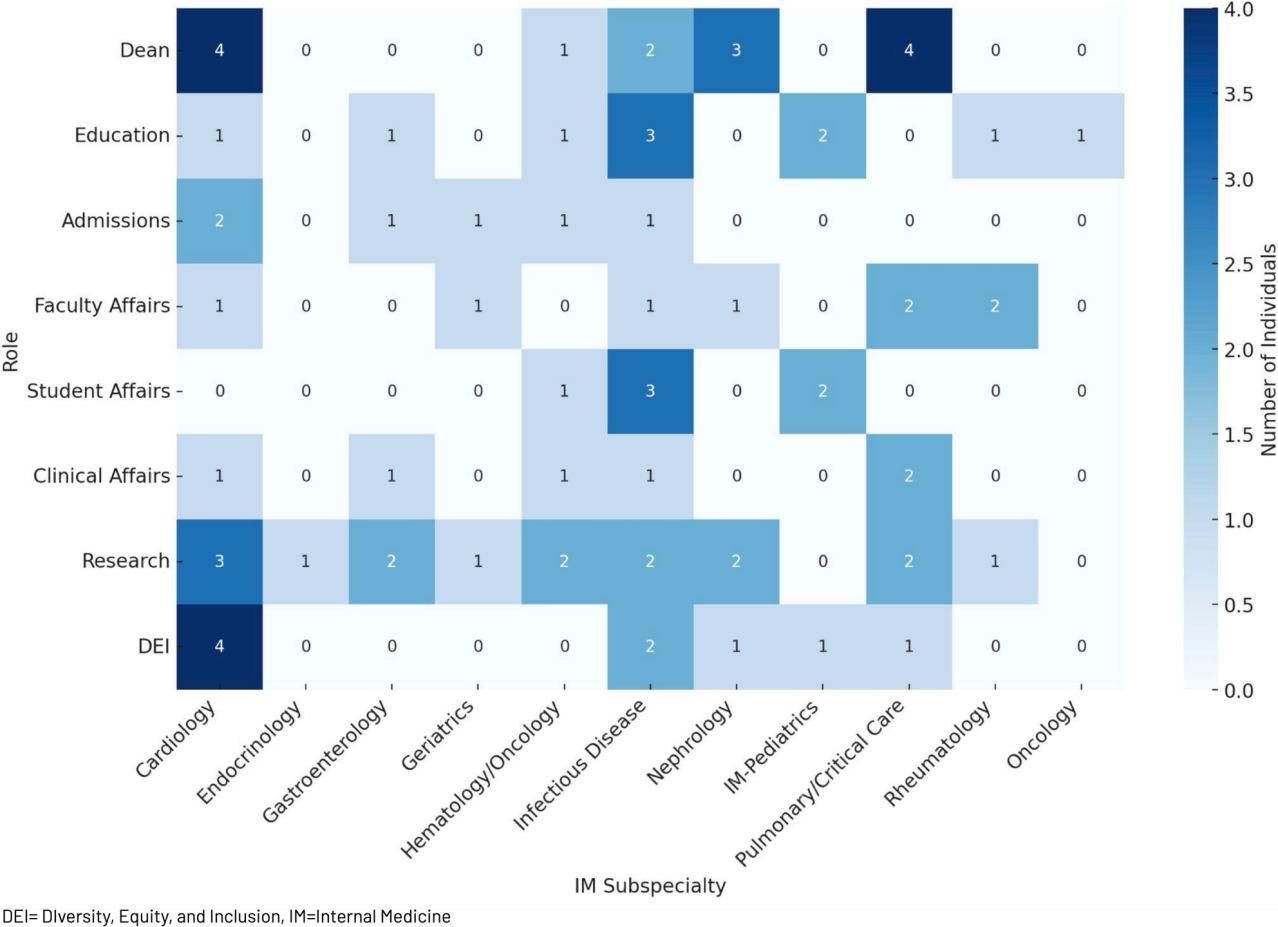
Heat map of represented internal medicine sub-specialties by dean’s cabinet role

Of the 128 IM Dean’s Cabinet members, 53 (41.4%) were trained in general internal medicine (non-subspecialty trained) while 75 (58.6%) belonged to various internal medicine subspecialties. The most represented IM subspecialty throughout all dean cabinet roles was Cardiology (16, 21.3%), followed by Infectious Disease (15, 20.0%) and Pulmonary/Critical Care (11, 14.7%). Cardiology (4, 5.3%) and Pulmonary/Critical Care (4, 5.3%) were the most common subspecialties to fulfill the position of Dean, Infectious Disease (3, 4.0%) for Dean of Student Affairs, Pulmonary/Critical Care (2, 2.7%) and Rheumatology (2, 2.7%) for Dean of Faculty Affairs, Pulmonary/Critical Care (2, 2.7%) for Dean of Clinical Affairs, Infectious Disease (3, 4.0%) for Dean of Education, Cardiology (3, 4.0%) for Dean of Research, Cardiology (2, 2.7%) for Dean of Admissions, and Cardiology (4, 5.3%) for Dean of Diversity, Equity and Inclusion. The distribution of IM subspecialties by dean’s cabinet role is further characterized in Fig. 2.

### Dean’s cabinet members holding other graduate degrees

Of the 33 dean’s cabinet members holding a graduate degree other than an MD or DO (e.g. PhD, MPH, MHA, EdD, MA), 25 (75.8%) held a PhD degree. 12 (36.4%) of these members were identified as a ‘Dean for Research.’

### Comparison to national workforce representation

**Fig. 3:**
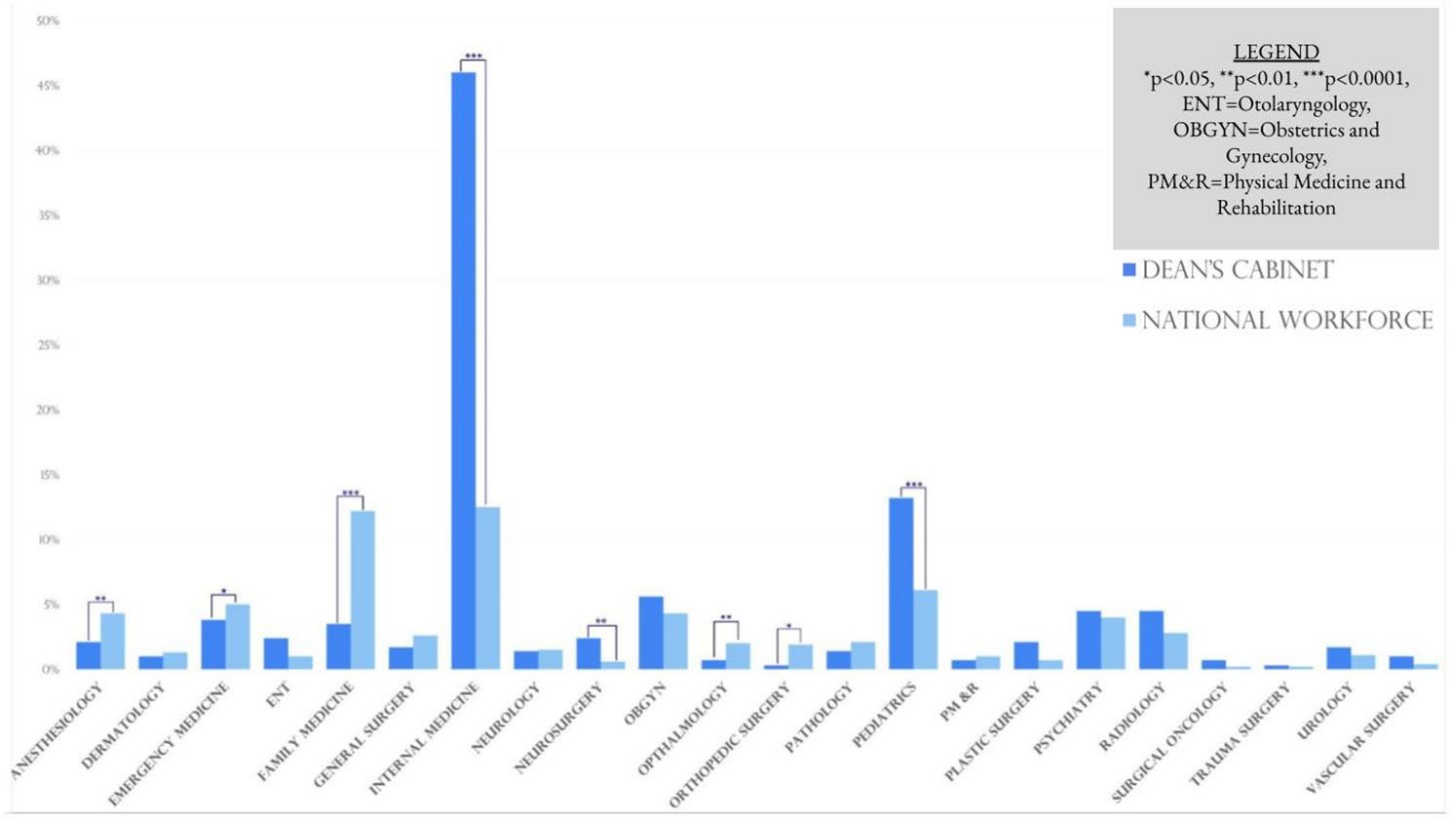
Comparison of represented specialties in dean’s cabinet to U.S. physician data

Compared to the U.S. Physician Workforce Data, Anesthesiology (*p* = 0.008), EM (*p* = 0.04), Orthopedic Surgery (*p* = 0.016), Ophthalmology (*p* = 0.037), and FM (*p* < 0.0001) were underrepresented while IM (*p* < 0.0001), Neurosurgery (*p* = 0.003), and Pediatrics (*p* = 0.006) were overrepresented in the dean’s cabinets of top 40 NIH funded medical schools. These relationships are further characterized in Fig. 3.

### Comparison to hospital based workforce representation

**Fig. 4:**
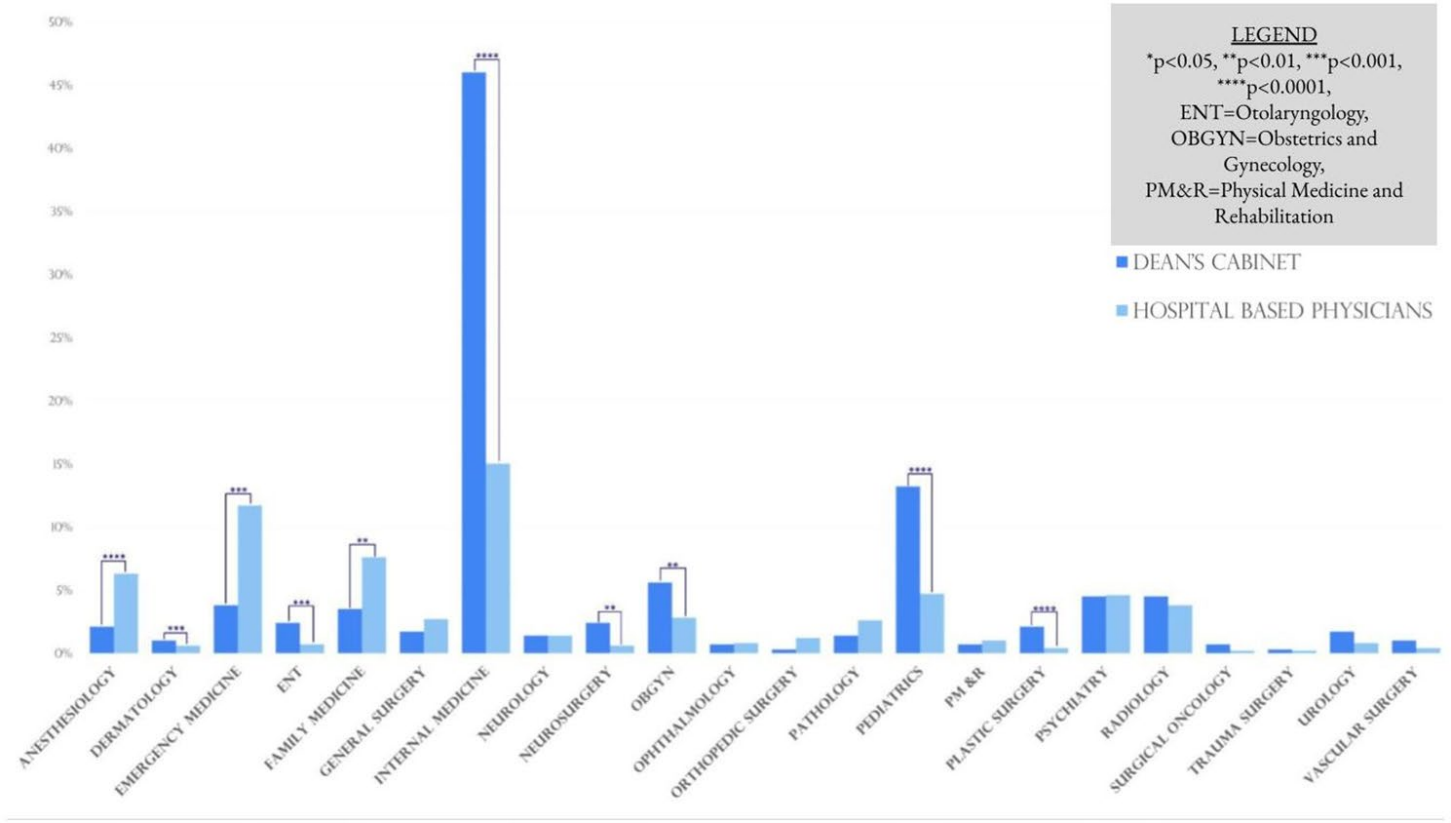
Comparison of represented specialties in dean’s cabinet to full-time hospital-based employees

Compared to the Hospital-Based Workforce Data, Anesthesiology (*p* < 0.0001), EM (*p* < 0.0001), and FM (*p* = 0.0084) were underrepresented while Dermatology (*p* = 0.0008), ENT (*p* = 0.0005), IM (*p* < 0.0001), Neurosurgery (*p* = 0.0001), OB/GYN (*p* = 0.0035), Pediatrics (*p* = 0.006), and Plastic Surgery (*p* < 0.00001) were overrepresented in the dean’s cabinets of top 40 NIH funded medical schools. These relationships are further characterized in Fig. 4.

## Discussion

This study reveals a striking concentration of medical school leadership in IM (132, 46.0%), Pediatrics (38, 13.2%), and OB/GYN (16, 5.6%). IM alone represents almost half (46%) of all dean’s cabinet members, an overrepresentation compared to the national physician workforce share (12.5%, *p* < 0.0001) as well as physicians working in a full-time hospital-based setting (15.0%, *p* < 0.00001) [5]. Notably, IM and pediatrics also consistently rank as the top two specialties across every cabinet role compared to national data. This pattern could be a reflection of physicians in primarily hospital-based specialties being more routinely involved in institutional operations, providing them with earlier and more frequent opportunities to engage in academic leadership. Moreover, IM and pediatrics are often regarded as the backbone of medicine, as both are generalist fields that emphasize holistic, longitudinal, and systems-based care [7]. This broad scope of practice may cultivate the adaptability, interdisciplinary collaboration, and institutional insight that are well-suited for leadership roles in academic medicine [8]. Concurrently, this concentration of leadership within a narrow set of specialties may influence institutional priorities through agenda-setting and strategic emphasis. Leadership composition can subtly shape student recruitment and advising, funding and resource allocation, and faculty appointment or promotion pathways in ways that reflect the professional norms of dominant fields, potentially reducing visibility and advocacy for underrepresented specialties despite existing governance checks and balances.

Interestingly, despite the critical roles that FM, EM, and Anesthesiology play in healthcare delivery, these specialties are significantly underrepresented among leadership in the dean’s cabinets of the nation’s top medical schools. FM and EM are both central to primary care, population health, and community engagement, and their representation (FM: 3.5%, EM: 3.8%) is notably scarce in comparison to the proportion of practicing FM and EM physicians in the nation (FM: 12.2%, EM: 5.0%) (*p* < 0.0001 and *p* < 0.05 respectively) as well as those employed in hospital-based settings (FM: 7.6%, EM: 11.7%) (*p* < 0.00001 and *p* < 0.0001 respectively). Similarly, anesthesiology, which plays an integral role in perioperative medicine and patient safety, is likewise underrepresented in medical leadership (2.1%) when compared to the proportion of practicing anesthesiologists in the nation (4.3%, *p* = 0.008) or hospital-based settings (6.3%, *p* < 0.00001). These findings carry important implications for institutional equity and the shaping of medical education priorities.

Underrepresentation of FM and EM is concerning given the national emphasis on addressing primary care shortages and health disparities especially in rural areas, and the rising amount of health misinformation available to the public [9, 10]. FM is uniquely positioned as a first line point of contact for patients, and plays a pivotal role in healthcare delivery, patient safety, and coordination of care, equipping patients with the knowledge and support needed to navigate complex health systems effectively. It is an integral pillar of medicine that needs to be appropriately represented in medical school leadership. The limited representation of FM physicians in leadership roles may reduce opportunities to incorporate perspectives that emphasize community-based care, interprofessional education, and interdisciplinary collaboration. Additionally, EM physicians serve as critical first-line providers who not only triage diverse clinical presentations but also play an essential role in medical education by training students and residents in acute care, clinical decision-making, and interdisciplinary teamwork. This disparity may offer insights on a general trend in which medical graduates pursuing hospital-based specialties characterized by longitudinal care express desire to engage in institutional leadership, whereas medical graduates entering fields like FM, EM or Anesthesiology may prioritize different professional goals and resultingly encounter fewer academic leadership opportunities [11]. 

Still, the disproportionate representation of certain fields in comparison to the national workforce (e.g. IM, Pediatrics, Neurosurgery) in this study raises questions of curricular rigidity, limiting the integration of emerging or cross-disciplinary specialties that are increasingly relevant to modern medicine, such as dermatology (1.0%) or ophthalmology (0.7%). For instance, underrepresented specialties including anesthesiology (2.1%) and EM (3.8%) benefit tremendously from educational modalities such as simulation-based learning and telehealth training [12, 13]. Additionally, fields such as radiology (4.5%) and dermatology (1.0%) have vast applications in primary care settings, yet remain a small portion of the dean’s cabinet leadership [14, 15]. This calls to attention the potentially negative impact of underrepresenting medical specialties that have broad applications touching all facets of modern medicine. Limited input from procedural or technology-driven fields such as these may delay the inclusion of simulation-based learning, telehealth training, or interdisciplinary team models in medical education. In this context, workforce comparisons serve to highlight system-level patterns in academic leadership rather than to suggest proportional specialty representation within individual institutions. Given the increasing complexity of patient care and the prevalence of comorbidities, exposure to academic mentors comprising a diverse range of clinical specialties and perspectives is essential to cultivating advanced critical thinking skills in medical students [16]. 

Compensation may be one factor influencing the over- or underrepresentation of certain specialties in medical school leadership. Fields such as IM or pediatrics are overrepresented medical specialties in deans’ cabinets, which may in part be explained by these leadership roles offering a financial incentive compared to their traditional practice with lower-than-average clinical salaries in comparison to other medical specialties [17]. In contrast, highly-compensated medical specialties such as anesthesiology, EM, or orthopedic surgery, which are underrepresented in leadership, may face a financial disincentive to pursue administrative roles that involve reduced clinical time and potentially lower overall compensation [17]. Interestingly, certain surgical fields such as neurosurgery and plastic surgery are overrepresented despite typically commanding some of the highest clinical salaries [18]. In these cases, the appeal of leadership may stem from other factors, such as influence over departmental priorities, academic advancement, or institutional prestige. This suggests that motivations for assuming administrative roles likely extend beyond compensation alone and may vary by specialty culture, career trajectory, and perceived impact within academic medicine.

Though this study focused on the clinical specialty as one dimension of academic leadership composition, leadership pathways in academic medicine are multidimensional. Factors such as degree type (e.g. MD, DO, PhD) and additional formal training in leadership, education, or administration play a substantial role in selecting members to represent medical institutions through senior leadership positions. Role-specific responsibilities may further influence which backgrounds are preferred for certain positions. For instance, research focused leadership roles (e.g. Dean for Research) may favor individuals with extensive grant funding or PhD training whereas education-focused roles may emphasize curricular expertise or longitudinal teaching experience. Predominantly clinical leadership may also have implications for pre-clinical and basic science education, as underrepresentation of PhD-trained faculty and pathologists in senior roles could influence how foundational science curricula are prioritized or resourced. These factors underscore that specialty representation reflects only one component of leadership diversity and should be interpreted within the broader context of institutional, professional, and structural influences.

### Limitations

This study has several limitations. First, focusing on the top 40 NIH-funded medical schools may introduce selection bias and limit generalizability, including the ability to assess alignment between leadership composition and institution-specific physician workforces. This analysis also excludes osteopathic, community-based, or rural medical schools, which may prioritize different leadership models and clinical specialties. Additionally, this is a cross-sectional study that relies on publicly available websites to identify dean’s cabinet members, allowing for the possibility of inaccuracies due to outdated or incomplete information, including the inclusion of interim or acting leadership appointments alongside permanent appointees. Finally, this study limited inclusion to the eight most common leadership titles, which may exclude other, institutionally specific, influential leadership roles and in turn underrepresent certain specialties that may be more likely to fill these less common roles.

## Conclusions

As medical education grapples with evolving population diversity, expanding scopes of practice, and increasing competition regarding the residency application process, equitable specialty representation at the leadership level is essential to ensure that the medical school curriculum is adaptable, inclusive, and forward-looking. Although this study highlights the disproportionate presence of certain specialties in academic leadership, further research is needed to assess how these imbalances may shape educational priorities, mentorship structures, and student career trajectories. Future studies could explore whether greater specialty diversity among leaders influences student career interests or promotes more comprehensive institutional decision-making. In response, medical schools might consider implementing structured leadership development programs for faculty in underrepresented specialties to foster more representative and well-rounded leadership teams.

## Data Availability

The datasets used and/or analyzed during the current study are available from the corresponding author on reasonable request. Data was gathered from information on publicly available institutional websites.

## Abbreviations

NIH: National Institutes of Health
AAMC: Association of American Medical Colleges
IM: Internal Medicine
OB/GYN: Obstetrics and Gynaecology
EM: Emergency Medicine
FM: Family Medicine
